# Single Port Robotic Surgery in Benign Urologic Disease – A Review of Contemporary Applications and Outcomes

**DOI:** 10.1007/s11934-026-01345-0

**Published:** 2026-07-04

**Authors:** Matthew J. Rabinowitz, Angeline Johny, Phillip Mucksavage

**Affiliations:** Division of Urology, University of Pennsylvania, 800 Walnut St. 19th Floor, Philadelphia, PA 19107 USA

**Keywords:** Single-Port Robotic Surgery, Benign Urology, Simple Prostatectomy, Robotic Pyeloplasty, Robotic Ureteral Reconstruction, Robotic Donor Nephrectomy

## Abstract

**Purpose of Review:**

The advent of the Intuitive da Vinci single-port (SP) robotic system represents a pivotal evolution in minimally invasive urologic surgery, offering enhanced dexterity, improved surgical ergonomics, and reduced patient morbidity. This review is designed to provide a comprehensive evaluation of current literature regarding the feasibility, efficacy, cost considerations, and clinical applications of the SP platform across various benign urologic surgeries including pyeloplasty, ureteral reconstruction, donor nephrectomy, renal auto-transplantation, partial cystectomy, and simple prostatectomy. The review also analyzes current limitations of the robotic platform including a narrower range of available instruments, diminished instrument grip strength, and a steeper learning curve.

**Recent Findings:**

Across these procedures, the SP system demonstrates comparable operative outcomes when compared to multi-port (MP) and open approaches. The SP system also has notable advantages in postoperative analgesia, incisional cosmesis, and hospital length of stay. The system’s low-profile design and ability to maintain an extraperitoneal approach confer distinct benefits in patients with complex surgical histories or hostile abdomens. Although the SP platform incurs higher disposable instrument costs, overall expenditures are mitigated with decreased hospital length of stay and reduced postoperative care requirements.

**Summary:**

As surgical experience and technological refinements progress, the SP robotic platform is anticipated to achieve broader integration in benign urology. Overall, SP robotic surgery offers a safe, effective, and patient-centered alternative to established minimally invasive techniques with the potential to further optimize perioperative outcomes and redefine the standard of care in contemporary urologic practice.

## Introduction

Since its adoption in the early 2000s, robotic assisted laparoscopic surgery has progressively transitioned into the first line approach for several urologic surgical interventions. Robotic approach has also been extensively investigated and is now recognized to have similar surgical outcomes when compared to open or laparoscopic approaches [1].

Robotic approach has undergone several evolutions and the most recent innovation, the Intuitive da Vinci single-port (SP) platform introduced in 2018, has challenged the paradigm of urologic surgical interventions yet again. The SP platform is the fourth generation of robotic surgical systems [2] and has advantages over the traditional multi-port platform including reduced surgical trauma, regionalizing surgical anatomy to avoid peritoneal entry, and as a result, enhancing post operative recovery [3]. Given the ability to remain extraperitoneal, the SP platform is a well-suited approach for patients with hostile abdomens from prior surgical history, those with concurrent colorectal disease, or from patients actively undergoing peritoneal dialysis [3]. Additionally, the low-profile nature of the SP robot confers greater versatility with surgical approach as well as a smaller footprint [3, 4]. However, limitations with the SP include decreased grip strength, less retraction ability, smaller selection of instruments, and a lack of advanced energy tools such as a vessel sealer or robotic stapler [3, 5, 6].

The objective of this review was to perform a comprehensive assessment of the existing literature regarding current feasibility, efficacy, and surgical approach as it pertains to the use of SP platform related to benign urologic conditions. We highlight the existing applications and review the potential future directions with this robotic platform.

## Methods

To perform this review, we queried PubMed Indexed articles published from 2019 to present relating to applications of SP robotic surgery in benign urologic disease and articles were not excluded based on specific study design requirements. Salient projects and topics were included at the discretion of the authors. Medical Subject Headings (MeSH) terms were used to specify our literature review, including but not limited to the following: Robotic Surgical Procedures; Ureteral diseases; Prostatectomy; Nephrectomies.

Additionally, our review seeks to review the early adoption and integration patterns of single site robotic surgery amongst urologists as well as examining cost-benefit analyses with respect to the use of the SP platform for benign urologic processes.

Following this, we categorized the articles based on relevant topics including pyeloplasty, ureteral reconstruction, donor nephrectomy/renal auto-transplantation, and simple prostatectomy, and reviewed existing literature for SP platform use treating these pathologies.

### Emergence and Adoption of SP Robotics

Prior to single port robotic surgery, the bridge between minimally invasive surgery and “scarless surgery” was single port laparoscopic surgery performed via the umbilicus. Most extirpative and reconstructive surgery was performed using this method, however, this technique led to significant challenges with camera clashing resulting in steep learning curves along with the need for curved, articulating instruments [7].

The introduction of the da Vinci robotic SP surgical system was able to bridge this gap by introducing “wristed” instruments that allowed for more precise articulation when compared to laparoscopic approaches and motion scaling along with tremor control [7]. The robotic SP platform sought to improve the ergonomics of single site surgery with the aim of wider utilization and ultimately, for enhanced patient recovery. In 2008, Kaouk and colleagues described the first successful cases of patients who had undergone single port trans-umbilical surgeries (radical prostatectomy, dismembered pyeloplasty, and radical nephrectomy). In his review, he describes feasibility using the single port platform along with improved aesthetics. Notably, the lower profile of the single port robot along with multiple flexible arms allows surgical progression without the restrictions of triangulation conventionally seen in laparoscopic surgery [7].

Historically, surgical outcomes between single site versus multi-port approach were investigated in older robotic platforms such the da Vinci S^®^ surgical system (Intuitive Surgical, Sunnyvale, CA, USA) [8]. In a retrospective review conducted by Rha and colleagues, laparoendoscopic single site (LESS) robotic partial nephrectomy outcomes were compared to those of conventional robotic partial nephrectomies. The single site group had longer warm ischemia and total operative times, however interestingly, similar rates of post op complications, negative margins, and changes in renal function. Additionally, the single site group had lower pain scores at discharge when compared to the conventional group [8].

These studies support early observations that single site surgery is a feasible modality with potentially similar functional post operative outcomes, along with added benefits of patient cosmesis and reduced pain during recovery.

## Utilization and Cost-Benefit

Surgical innovation focuses on improving patient care, yet wider application can be limited by cost. Analyzing the economy of utilizing new technology is critical to assess for broader utilization. In a study by Kaouk and colleagues from the Cleveland Clinic, surgical care costs were retrospectively compared between single port and multi-port cohorts who underwent robotic radical prostatectomies [9]. Unlike multi-port surgery, single port surgical instruments are mainly disposable, thus incurring higher operative costs. Interestingly, the mean costs between the two groups were similar despite this, due to significantly shorter length of hospitalization in the single port group when compared to the multi-port group [8].

The mean total cost between both groups did not differ significantly, nor did the initial purchase or maintenance of the two robotic surgical platforms [9]. As expected, the disposable cost of surgical instruments in the single port group was significantly higher, ranging from $1349-$3541, compared to the multi-port group cost ranging from $453-$1073. Anesthesia and OR utilization costs were similar between both groups, and pharmacy costs were lower among the single port group, highlighting the decreased need of post operative medication such as narcotics in this cohort [9].

In a similar study led by Crivellaro and colleagues, an SP robotic radical prostatectomy patient cohort was compared to MP robotic radical prostatectomy, and both had similar operative times, similar rate of complications, and similar functional outcomes, yet the SP group had lower median length of stay and greater proportion of patients reporting they were pain free [10]. Another multi-disciplinary meta-analysis comprising of data from general surgery, urology, and gynecology reviewed outcomes between MP and SP procedures [11]. From a urologic standpoint, SP procedures were noted to have a significant reduction in post operative pain, better cosmetic outcomes, and reduction in instrument clashing [11].

In fact, studies have investigated pain differences between the two robotic platforms and have found that patients having undergone SP robotic procedures had less pain, and decreased use of narcotics. In a single institution, retrospective review of patients who had undergone robotic assisted simple prostatectomy, the single port group had a nearly 50% decrease in narcotic usage [12]. In another review investigating post operative pain among partial nephrectomy patients, multiple studies highlighted that a major advantage of the SP system was decreased postoperative pain, which theoretically avoids the need for post operative opioids [13].

These articles again reinforce feasibility of this robotic platform and demonstrate comparable healthcare costs given advantages of decreased post operative pain and faster recoveries leading to expedited discharges. Surgical innovation is often a double-edged sword, with newer technology carrying additional economic health burden. However, the decreased costs of ancillary services in SP post operative care, such as hospital length of stay and narcotic requirements, favors the SP platform.

## Pyeloplasty

Single port robotic pyeloplasty (SP-RP) represents a significant advancement in minimally invasive urologic surgery. The essential reconstructive steps of pyeloplasty—dissection of the ureteropelvic junction (UPJ), excision of the stenotic segment, spatulation of the ureter, and creation of a tension-free, watertight anastomosis—are fundamentally similar between SP and MP approaches. Stent placement and drain use are at the surgeon’s discretion, but SP-RP often obviates the need for routine drain placement, further reducing invasiveness [14, 15]. The workflow of SP-RP, including patient positioning, access, and docking, is designed to maximize cosmesis, minimize tissue trauma, and enable same-day discharge protocols [16].

Comparative studies and meta-analyses demonstrate that SP-RP achieves perioperative and long-term outcomes equivalent to MP-RP in both adult and pediatric populations. In a propensity-score matched analysis of adults, SP-RP was associated with a longer median operative time compared to MP-RP (128.0 vs. 88.0 min, *P* = 0.0411), though this difference was only marginally significant in sensitivity analysis (*P* = 0.0540) [17] .However, a meta-analysis of five retrospective cohort studies (*n* = 179) found no statistically significant difference in operative time between SP-RP and MP-RP, and a separate meta-analysis reported that SP-RP may reduce operative time for pyeloplasty (mean difference − 0.73, 95% CI −1.24 to −0.22) as experience accrues [18, 19]. Complication rates are low and comparable between SP-RP and MP-RP, with no significant differences in intraoperative or postoperative complications in adults or children [16, 17, 20, 21].

Success rates for both SP-RP and MP-RP is consistently higher than 90%, as measured by symptom resolution and radiographic improvement on follow-up imaging. For example, in a pediatric cohort of 327 patients, radiographic improvement was observed in 94.2% of patients at long-term follow-up, with most failures identified within the first year postoperatively and no recurrences beyond three years [22]. In adults, long-term follow-up studies of robotic pyeloplasty (primarily MP-RP) report failure-free survival rates exceeding 90% at 8 years, and early data for SP-RP suggest similar outcomes [23]. Hospital stay is consistently shorter with SP-RP, with same-day discharge being feasible in most cases (mean length of stay 11.4 h for SP-RP vs. 42.6 h for MP-RP, *P* < 0.001) [14–16, 19]. Postoperative pain is reduced with SP-RP, as reflected by lower opioid usage in the hospital (*P* < 0.001) and at discharge (18.2% vs. 91.7%, *P* < 0.001), and pain management can often be achieved exclusively with non-opioid medications [1–3, 5]. Cosmetic outcomes are superior with SP-RP, as the single, often hidden incision results in minimal visible scarring.

Nonetheless, the learning curve is steeper than for SP-RP when compared to MP-RP, with longer operative times during the initial phase of adoption. The lack of natural triangulation and the coaxial alignment of instruments can make certain steps, such as retraction and exposure, more difficult, particularly in cases with significant peripelvic fat, aberrant vasculature, or complex anatomy. Proficiency is typically achieved after 13–18 cases and mastery after 30–40 cases, as demonstrated in multiple pediatric series [20, 24].

The current evidence base for SP-RP is limited by small sample sizes, retrospective study designs, and relatively short follow-up in most comparative studies. There is a lack of large-scale, multicenter, prospective studies and randomized controlled trials directly comparing SP-RP and MP-RP, and most meta-analyses aggregate data from heterogeneous procedures and patient populations. Structured training, careful patient selection, and institutional support are essential for successful adoption and outcomes.

## Ureteral Reconstruction

Single-port robotic surgery for ureteral reconstruction allows for this complex reconstructive procedure to be performed through a single infra-umbilical incision. Stent placement during reconstruction is performed in many described procedures percutaneously prior to completion of the anastomosis, using a 14-gauge angiocatheter for guidance. The technique allows for both refluxing and non-refluxing ureteroneocystostomy, depending on the clinical scenario. Adjuncts such as near-infrared fluorescence and concurrent ureteroscopy have been described to aid in identification of the ureter and assessment of tissue perfusion, particularly in complex cases such as post-transplant strictures, where preservation of vascular supply is critical [25–27].

Early analysis of perioperative outcomes demonstrated that single-port robotic ureteral reconstruction offers efficiency and safety comparable to, and in some studies superior to, traditional multi-port robotic and laparoscopic approaches. Kaouk et al. reported mean operative times of 165, 150, and 180 minutes for three consecutive SP cases, with consistently minimal (< 50mL) blood loss and no notable intraoperative complications. All patients were discharged on postoperative days 1 or 2, with normal serum creatinine levels and no major complications [25]. Hebert et al. described a single-port ureteroneocystostomy completed in 127 minutes with an estimated blood loss of 20 cc and no complications, highlighting the technical feasibility and safety of the SP approach [26].

In pediatric populations, Lin et al. compared single-port-plus-one (an additional assistant port is placed alongside the traditional single-port globe) robotic and traditional laparoscopic approaches for primary obstructive megaureter. Of note, the former relied on the da Vinci Xi system introduced through a quadruple-channel puncture device. Both techniques achieved 100% obstruction resolution, but the SP-plus-one approach was associated with significantly less blood loss, gross hematuria time, indwelling catheterization time, and hospitalization time. The total operation time in the SP group was slightly longer, but intraperitoneal operation time was comparable. No unique adverse events were reported, and complication rates were low [28].

Heo et al. describe their experience with single port robotic ureteral reimplantation in a series of 21 patients. The median operative time was 152 minutes (84–354). The median blood loss was 10 mL (5–330 mL), and the median length of stay in hospital was 4 days (2–10 d). One patient in this cohort required a conversion to an open approach as a result of a serosal injury during lysis of adhesions and one patient required nephrostomy tube placement on post-operative day 2 as a result of stent malposition. The rates of radiographic and symptomatic improvement at 3 months were both 100% in this group [29]. Additionally, Kaouk et al. reported in their case series normal serum creatinine levels in the peri-operative period and no major complications at time of discharge, though they did not provide extended follow-up data [25]. Chen et al. described three cases of transplant ureteral stricture managed with SP robotic reconstruction, all of which resulted in resolution of hydronephrosis and allograft dysfunction, with no reported complications during follow-up [30].

These findings, while limited in scope, indicate high rates of radiographic and symptomatic resolution following SP robotic reconstruction in both adult and pediatric populations, though longer-term data and larger cohorts are needed to fully assess durability. Nonetheless, single-port robotic surgery for ureteral reconstruction is a technically feasible and safe approach in both adult and pediatric populations, with perioperative advantages including reduced pain, improved cosmesis, shorter hospital stays, and comparable complication rates to traditional multi-port and laparoscopic techniques.

## Partial Cystectomy/Bladder Diverticulectomy

Single-port robotic bladder diverticulectomy is an emerging technique that has been described in a limited number of case reports and series. Both transvesical and transperitoneal approaches have been described. As described by Roslan and colleagues, the transvesical approach involves direct access to the bladder through a small suprapubic incision, allowing for precise excision and closure with minimal blood loss, short catheterization, and rapid recovery; early clinical experiences report favorable outcomes and low complication rates in both male and female patients [31, 32]. The transperitoneal single-port robotic approach, including Firefly-guided techniques, further enhances visualization and can be combined with procedures such as simple prostatectomy, offering a versatile platform for complex cases [33].

Transvesical and transabdominal (extravesical) approaches should be individualized, with transvesical entry providing direct visualization and protection of adjacent structures, while the extravesical route may be preferable for certain anatomical scenarios or when concomitant treatment of bladder outlet obstruction is required [34]. Across reported series, operative times are reasonable (120–140 minutes), blood loss is minimal, and hospital stays are short, with most patients experiencing significant symptomatic improvement and low rates of perioperative complications [31, 32, 34].

### Renal transplant/Auto-transplantation

In the context of kidney transplants and auto-transplantation, minimally invasive approaches have been gaining increasing popularity due to decreased wound complications as well as improved post operative recovery time [35, 36]. Using the SP approach to this end was first described by Kaouk and colleagues, with both transperitoneal and extraperitoneal techniques [35]. This case series provided a detailed overview of surgical technique as well as a description of post operative course. All six patients in the study had operative times ranging from 300 to 450 minutes, comparable to open counterparts, in addition to well perfused allografts without hydronephrosis on Doppler ultrasound [35]. Impressively, only one patient required narcotics (1.6 morphine milligram equivalents) and the average hospital stay was 2–3 days [35]. In another meta-analysis reviewing robotic kidney transplants, including in obese patients, the overall findings of robotic assisted kidney transplant has lower incidence of surgical complications and similar graft survival outcomes [37].

## Donor Nephrectomy

The drive for minimally invasive approaches for donor nephrectomies were rooted in desire for early post operative recovery and early return to daily activities [38]. A meta-analysis by Papalois and colleagues compared outcomes between laparoscopic versus open transplant nephrectomies and found that the laparoscopic approach had significantly lower total blood loss, lower rates of analgesic requirements, and no difference in graft loss at 1 year [38]. Robotic donor nephrectomies have also been evaluated for safety and feasibility. In a case series at a single institution led by Levy et al., donor multi-port and single port robotic donor nephrectomies had comparable outcomes including operative times, bleeding, and allograft function [39]. Palese and colleagues retrospectively reviewed outcomes of patients who had undergone laparoscopic donor nephrectomies and single port donor nephrectomies, finding similar outcomes in complications, post operative opioid usage, and renal allograft function [40]. These studies reiterate the central principles of single port surgery: preserved surgical outcomes, expedited surgery, with a decrease in expected post operative pain. One single institution retrospective review and surgical technique video, led by Levy and collages, is comprised of 51 donor patients, and cites several advantages of the single port robotic approach [41]. Among these advantages are improved maneuverability, visibility of the renal hilum, especially in the context of complex cases, with improved cosmetic outcomes. The SP approach also allows for greater degrees of freedom with complex vascular dissection, which is especially critical for donor nephrectomies [41]. Despite its many advantages, SP donor nephrectomies have not been widely adopted.

The benign applications of SP robot are vast, but especially meaningful in altruistic surgeries such as donor nephrectomies, affording surgeons improved visualization and patients improved recovery and cosmetic outcomes.

## Simple Prostatectomy

Single-port prostatectomy (SPP), first introduced within the past decade, has become increasingly adopted at robotic centers of excellence, although long-term outcome data remains limited. Both transvesical and extraperitoneal approaches have demonstrated favorable perioperative outcomes, including shorter hospitalizations, improved postoperative pain control, and reduced narcotic utilization [12, 42, 43]. Moreover, a systematic review published in 2024 including 160 patients reported improved pain outcomes, lower estimated blood loss, and comparable complication rates among the SP simple prostatectomy cohort when comparing to existing approaches [44].

Similarly, the largest single-institution cohort study to date, published by the Cleveland Clinic in 2024, supports these findings and notes that patients undergoing SP simple prostatectomy had minimal blood loss, and excellent post operative outcomes, with greater than 95% same-day discharge rates [43]. Comparative analyses of multi-port versus single-port prostatectomy have shown no significant differences in perioperative outcomes or operative times, which are anticipated to improve with increasing surgeon experience [45]. Conversely, a review in outcomes between open and single-port approaches demonstrated a clear advantage in the single port cohort [46]. A small series has even reported the feasibility of performing single-port trans vesical simple prostatectomy under regional anesthesia alone, underscoring the versatility of this evolving technique and broader application of this surgery for patients who may not be appropriate for general anesthesia [47].

The existing literature supports single port simple prostatectomy as a surgical approach with clear advantages in patient recovery, decreased blood loss, and post operative outcomes when compared to traditional techniques. As SP adoption expands, and surgeon experience improves, single port simple prostatectomy is expected to increase in adoption in contemporary urology practice.

## Conclusions

The single port robot is the fourth-generation robotic platform that has created a paradigm shift in surgical applications, patient recovery, and anticipated hospital stays [6]. In comparing single port with multi-port, the SP group has higher operating room costs due to disposable instruments, yet this is offset with decreased hospital costs from shorter length of stays [6]. Multiple studies have supported ease of patient recovery and improved post operative pain, underscoring a key advantage of this minimally invasive technique. Another key advantage is the maneuverability of instruments, especially within confined spaces. A smaller working footprint allows surgeons to operate within limited regions. However, this lends itself to steeper learning curves, as this is a stark shift from the spread-out working space of instruments in multi-port laparoscopic and robotic surgery. Additionally, the SP system has reduced grip strength requiring the surgeon to approach dissection and retraction using different techniques, not often utilized in multi-port surgery. Within multiple domains of benign robotic urologic conditions, single port surgery has demonstrated feasibility, safety, improved recovery, and enhanced visibility. Limitations are focused on the learning curve involved with learning the robotic platform and the restrained working space.

As single-port surgery garners more momentum and gains widespread adoption, newer training models and improved instruments are imperative to blunt existing steep surgeon learning curves. Nonetheless, the SP platform already confers several advantages that make it a valuable tool in the urologist’s armamentarium, especially in the context of benign urologic conditions.

## Key References

- Khalil MI, Chase A, Joseph JV, Ghazi A. Standard Multiport vs Single-Port Robot-Assisted Simple Prostatectomy: A Single-Center Initial Experience. J Endourol. 2022;36(8):1057-1062. doi:10.1089/end.2021.0510 No difference in surgical outcomes between SP and MP simple prostatectomy
- Nguyen TT, Basilius J, Ali SN, Dobbs RW, Lee DI. Single-Port Robotic Applications in Urology. *Journal of Endourology*. 2023;37(6):688-699. doi:10.1089/end.2022.0600 Overview of future directions for the SP platorm

## Data Availability

No datasets were generated or analysed during the current study.

## Declarations

The article does not contain any studies with human or animal subjects performed by any of the authors.
